# 4-Hydr­oxy-4,4-diphenyl­butan-2-one

**DOI:** 10.1107/S1600536808020886

**Published:** 2008-07-12

**Authors:** Dennis P. Arnold, John C. McMurtrie

**Affiliations:** a School of Physical and Chemical Sciences, Queensland University of Technology, 2 George St, Brisbane, Queensland 4001, Australia

## Abstract

The mol­ecules of the title compound, C_16_H_16_O_2_, display an intra­molecular O—H⋯O hydrogen bond between the hydroxyl donor and the ketone acceptor. Inter­molecular C—H⋯π inter­actions connect adjacent mol­ecules into chains that propagate parallel to the *ac* diagonal. The chains are arranged in sheets, and mol­ecules in adjacent sheets inter­act *via* inter­molecular O—H⋯O hydrogen bonds.

## Related literature

For related literature, see: Rivett (1980 ▶); Paulson *et al.* (1973 ▶).
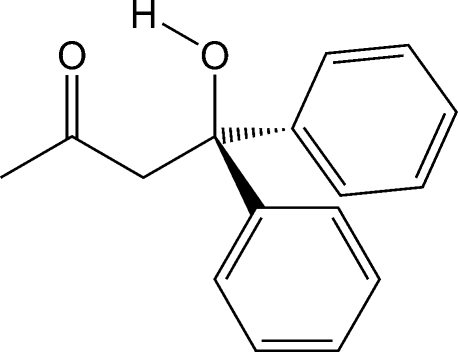


## Experimental

### 

#### Crystal data

C_16_H_16_O_2_
*M* *_r_* = 240.29
Monoclinic, 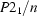

*a* = 9.8619 (2) Å
*b* = 9.2015 (2) Å
*c* = 14.3720 (3) Å
β = 102.098 (2)°
*V* = 1275.21 (5) Å^3^
*Z* = 4
Mo *K*α radiation
μ = 0.08 mm^−1^
*T* = 150 (2) K
0.35 × 0.30 × 0.20 mm

#### Data collection

Oxford Diffraction Gemini diffractometer
Absorption correction: multi-scan (*CrysAlis RED*; Oxford Diffraction, 2007 ▶) *T*_min_ = 0.933, *T*_max_ = 0.984
7361 measured reflections
2935 independent reflections
2144 reflections with *I* > 2σ(*I*)
*R*_int_ = 0.021

#### Refinement

*R*[*F* ^2^ > 2σ(*F* ^2^)] = 0.036
*wR*(*F* ^2^) = 0.066
*S* = 1.01
2935 reflections
167 parameters
1 restraint
H atoms treated by a mixture of independent and constrained refinement
Δρ_max_ = 0.22 e Å^−3^
Δρ_min_ = −0.20 e Å^−3^

### 

Data collection: *CrysAlis CCD* (Oxford Diffraction, 2007 ▶); cell refinement: *CrysAlis RED* (Oxford Diffraction, 2007 ▶); data reduction: *CrysAlis RED*; program(s) used to solve structure: *SIR97* (Altomare *et al*. 1999 ▶); program(s) used to refine structure: *SHELXL97* (Sheldrick, 2008 ▶); molecular graphics: *ORTEP-3* (Farrugia, 1997 ▶) and *CrystalMaker* (CrystalMaker, 2006 ▶); software used to prepare material for publication: *publCIF* (Westrip, 2008 ▶).

## Supplementary Material

Crystal structure: contains datablocks I, global. DOI: 10.1107/S1600536808020886/hg2422sup1.cif

Structure factors: contains datablocks I. DOI: 10.1107/S1600536808020886/hg2422Isup2.hkl

Additional supplementary materials:  crystallographic information; 3D view; checkCIF report

## Figures and Tables

**Table 1:** Hydrogen-bond geometry (Å, °)

*D*—H⋯*A*	*D*—H	H⋯*A*	*D*⋯*A*	*D*—H⋯*A*
O2—H2*O*⋯O1	0.910 (12)	2.016 (12)	2.7636 (12)	138.5 (11)
O2—H2*O*⋯O1^i^	0.910 (12)	2.385 (13)	3.0530 (12)	130.3 (10)

Symmetry code: (i) 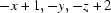.

**Fig. 1.**
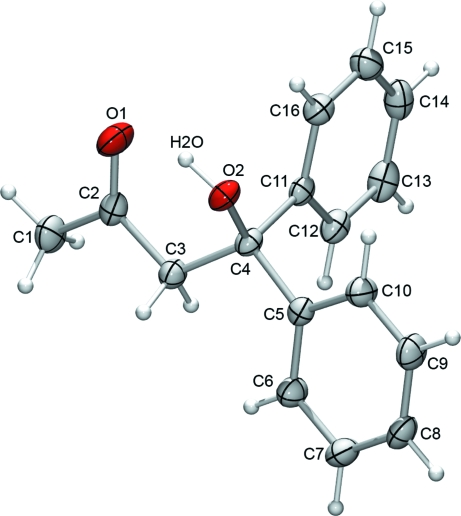
ORTEP depiction of the molecular structure with atom numbering scheme. Ellipsoids are drawn at the 50% probability level.

**Fig. 2.**
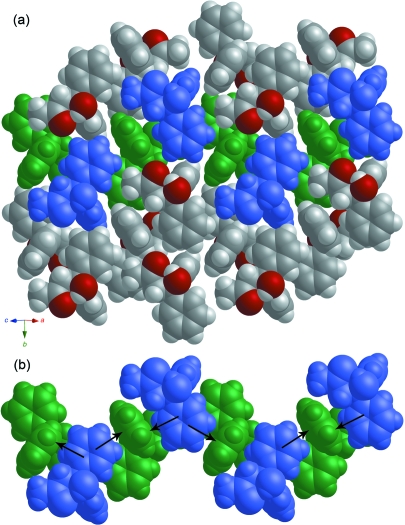
(a) The molecules are arranged in sheets. Within the sheets the molecules are linked in one-dimensional chains by CH···π interactions between phenyl rings. Once such chain is highlighted with alternating molecules coloured green and blue. (b) Excerpt from (a) showing the propagation of the chain by CH···π interactions between phenyl rings of adjacent molecules.

**Fig. 3.**
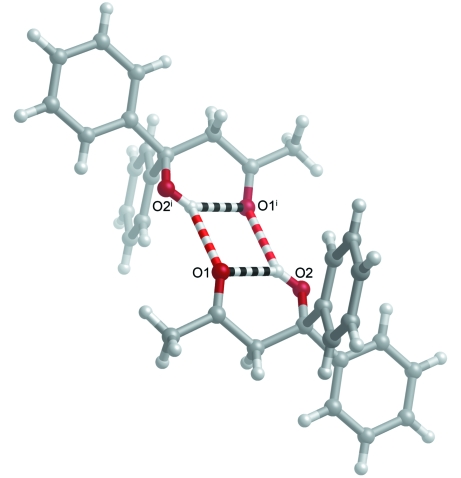
Molecules in adjacent two-dimensional sheets are connected by intermolecular hydrogen bonds. The arrangement of these hydrogen bonds between a pair of molecules is illustrated (red/white dashed contact). The intramolecular hydrogen bonds are also shown (black/white dashed line). Symmetry code: (i) -x + 1, -y, -z + 2.

## supplementary crystallographic information

### Comment

The molecular stucture of the title compound, (I), is illustrated in Fig. 1. There is an intramolecular hydrogen bond between the hydroxyl moiety and the ketone oxygen. The molecules are arranged in chains that propagate parallel to the *ac* diagonal *via* intermolecular CH···π interactions. There are two such interactions; the aromatic ring comprising C5—C10 is the CH donor and the ring comprising C11—C16 is the acceptor in both cases. Geometric parameters for the two interactions are as follows; C6—H6···C11—C16_plane_ distance 2.731 Å with C5—C10_plane_···C11—C16_plane_ dihedral angle 78.67° and C8—H8···C11—C16_plane_ distance 2.947 Å with C5—C10_plane_···C11—C16_plane_ dihedral angle 83.52°. The chains stack to form two-dimensional sheets in the crystal structure (Fig. 2). Intermolecular hydrogen bonds connect pairs of molecules from contiguous two-dimensional sheets. The pairwise intermolecular H-bond interactions and the intramolecular H-bond interactions are illustrated in Fig. 3.

### Experimental

The title compound was prepared according to the procedure described by Rivett (1980) which is an adaptation of the method reported earlier by Paulson *et al.* (1973). Large colourless prismatic crystals of the compound were obtained by crystallization from an evaporating dichloromethane/methanol solution.

### Refinement

C-bound H atoms were included in idealized positions and refined using a riding model approximation with methylene, methyl and aromatic bond lengths fixed at 0.99, 0.98 and 0.95 Å, respectively. *U*_iso_(H) values were fixed at 1.2*U*_eq_ of the parent C atoms for methylene and aromatic H atoms and 1.5*U*_eq_ of the parent C atoms for methyl H atoms. The hydroxy H atom was located in a Fourier difference map and refined with an O—H bond length restraint of 0.98 Å and with *U*_iso_ fixed at 1.5*U*_eq_ of the parent O atom.

### Crystal data

**Table:**

C_16_H_16_O_2_	*F*_000_ = 512
*M**_r_* = 240.29	*D*_x_ = 1.252 Mg m^−^^3^
Monoclinic, *P*2_1_/*n*	Mo *K*α radiation λ = 0.71073 Å
Hall symbol: -P 2yn	Cell parameters from 3618 reflections
*a* = 9.8619 (2) Å	θ = 2.9–28.7º
*b* = 9.2015 (2) Å	µ = 0.08 mm^−^^1^
*c* = 14.3720 (3) Å	*T* = 150 (2) K
β = 102.098 (2)º	Prism, colourless
*V* = 1275.21 (5) Å^3^	0.35 × 0.30 × 0.20 mm
*Z* = 4	

### Data collection

**Table:**

Oxford Diffraction Gemini diffractometer	2935 independent reflections
Radiation source: Enhance (Mo) X-ray Source	2144 reflections with *I* > 2σ(*I*)
Monochromator: graphite	*R*_int_ = 0.021
Detector resolution: 16.0774 pixels mm^-1^	θ_max_ = 28.8º
*T* = 150(2) K	θ_min_ = 2.9º
ω scans	*h* = −11→12
Absorption correction: multi-scan(CrysAlis RED; Oxford Diffraction, 2007)	*k* = −12→12
*T*_min_ = 0.933, *T*_max_ = 0.984	*l* = −19→19
7361 measured reflections	

### Refinement

**Table:**

Refinement on *F*^2^	Secondary atom site location: difference Fourier map
Least-squares matrix: full	Hydrogen site location: inferred from neighbouring sites
*R*[*F*^2^ > 2σ(*F*^2^)] = 0.036	H atoms treated by a mixture of independent and constrained refinement
*wR*(*F*^2^) = 0.066	*w* = 1/[σ^2^(*F*_o_^2^) + (0.01*P*)^2^ + 0.4*P*] where *P* = (*F*_o_^2^ + 2*F*_c_^2^)/3
*S* = 1.01	(Δ/σ)_max_ = 0.001
2935 reflections	Δρ_max_ = 0.22 e Å^−^^3^
167 parameters	Δρ_min_ = −0.20 e Å^−^^3^
1 restraint	Extinction correction: none
Primary atom site location: structure-invariant direct methods	

### Special details

**Table:**

Experimental. Crystal cleaved from larger prism.Empirical absorption correction using spherical harmonics, implemented in SCALE3 ABSPACK scaling algorithm.
Geometry. All e.s.d.'s (except the e.s.d. in the dihedral angle between two l.s. planes) are estimated using the full covariance matrix. The cell e.s.d.'s are taken into account individually in the estimation of e.s.d.'s in distances, angles and torsion angles; correlations between e.s.d.'s in cell parameters are only used when they are defined by crystal symmetry. An approximate (isotropic) treatment of cell e.s.d.'s is used for estimating e.s.d.'s involving l.s. planes.
Refinement. Refinement of *F*^2^ against ALL reflections. The weighted *R*-factor *wR* and goodness of fit *S* are based on *F*^2^, conventional *R*-factors *R* are based on *F*, with *F* set to zero for negative *F*^2^. The threshold expression of *F*^2^ > σ(*F*^2^) is used only for calculating *R*-factors(gt) *etc*. and is not relevant to the choice of reflections for refinement. *R*-factors based on *F*^2^ are statistically about twice as large as those based on *F*, and *R*- factors based on ALL data will be even larger.

### Fractional atomic coordinates and isotropic or equivalent isotropic displacement parameters (Å^2^)

**Table:**

	*x*	*y*	*z*	*U*_iso_*/*U*_eq_	
C1	0.55546 (14)	0.14657 (15)	0.76771 (9)	0.0373 (3)	
H1A	0.6047	0.0538	0.7694	0.056*	
H1B	0.6224	0.2266	0.7750	0.056*	
H1C	0.4896	0.1562	0.7067	0.056*	
C2	0.47849 (12)	0.15136 (14)	0.84736 (8)	0.0276 (3)	
C3	0.42255 (12)	0.29752 (13)	0.86860 (8)	0.0256 (3)	
H3A	0.3802	0.3449	0.8076	0.031*	
H3B	0.5012	0.3589	0.9004	0.031*	
C4	0.31411 (11)	0.29285 (13)	0.93164 (7)	0.0224 (2)	
C5	0.27448 (11)	0.44508 (13)	0.95954 (8)	0.0227 (2)	
C6	0.30596 (13)	0.57091 (14)	0.91585 (8)	0.0294 (3)	
H6	0.3561	0.5648	0.8663	0.035*	
C7	0.26527 (13)	0.70651 (14)	0.94343 (9)	0.0328 (3)	
H7	0.2875	0.7920	0.9127	0.039*	
C8	0.19261 (12)	0.71662 (14)	1.01542 (8)	0.0311 (3)	
H8	0.1649	0.8089	1.0345	0.037*	
C9	0.16028 (13)	0.59169 (15)	1.05965 (9)	0.0330 (3)	
H9	0.1100	0.5983	1.1091	0.040*	
C10	0.20081 (13)	0.45708 (14)	1.03224 (8)	0.0291 (3)	
H10	0.1783	0.3719	1.0632	0.035*	
C11	0.18229 (11)	0.21520 (13)	0.87881 (7)	0.0221 (2)	
C12	0.10703 (12)	0.27142 (14)	0.79318 (8)	0.0282 (3)	
H12	0.1379	0.3576	0.7677	0.034*	
C13	−0.01221 (13)	0.20283 (15)	0.74497 (8)	0.0336 (3)	
H13	−0.0627	0.2423	0.6869	0.040*	
C14	−0.05786 (13)	0.07704 (15)	0.78118 (9)	0.0344 (3)	
H14	−0.1390	0.0294	0.7477	0.041*	
C15	0.01500 (13)	0.02096 (15)	0.86617 (9)	0.0341 (3)	
H15	−0.0165	−0.0650	0.8915	0.041*	
C16	0.13418 (12)	0.08994 (14)	0.91473 (8)	0.0281 (3)	
H16	0.1834	0.0508	0.9733	0.034*	
O1	0.46398 (10)	0.04232 (10)	0.89213 (6)	0.0388 (2)	
O2	0.37110 (8)	0.22230 (9)	1.01953 (5)	0.02635 (19)	
H2O	0.4104 (14)	0.1379 (14)	1.0054 (9)	0.040*	

### Atomic displacement parameters (Å^2^)

**Table:**

	*U*^11^	*U*^22^	*U*^33^	*U*^12^	*U*^13^	*U*^23^
C1	0.0371 (8)	0.0363 (8)	0.0426 (7)	0.0045 (6)	0.0178 (6)	−0.0073 (6)
C2	0.0246 (6)	0.0290 (7)	0.0283 (6)	0.0047 (5)	0.0030 (5)	−0.0038 (5)
C3	0.0260 (6)	0.0247 (6)	0.0271 (6)	0.0035 (5)	0.0077 (5)	−0.0001 (5)
C4	0.0258 (6)	0.0221 (6)	0.0196 (5)	0.0044 (5)	0.0057 (4)	0.0023 (5)
C5	0.0219 (6)	0.0225 (6)	0.0230 (5)	0.0026 (5)	0.0032 (4)	−0.0014 (5)
C6	0.0337 (7)	0.0256 (7)	0.0315 (6)	0.0036 (5)	0.0127 (5)	0.0010 (6)
C7	0.0379 (7)	0.0221 (6)	0.0391 (7)	0.0039 (6)	0.0099 (6)	0.0023 (6)
C8	0.0316 (7)	0.0245 (7)	0.0360 (6)	0.0080 (6)	0.0042 (5)	−0.0057 (6)
C9	0.0348 (7)	0.0341 (7)	0.0331 (6)	0.0062 (6)	0.0141 (5)	−0.0043 (6)
C10	0.0336 (7)	0.0259 (7)	0.0304 (6)	0.0016 (5)	0.0126 (5)	0.0008 (5)
C11	0.0247 (6)	0.0227 (6)	0.0200 (5)	0.0055 (5)	0.0074 (4)	−0.0024 (5)
C12	0.0318 (6)	0.0310 (7)	0.0227 (6)	0.0070 (6)	0.0078 (5)	0.0012 (5)
C13	0.0318 (7)	0.0444 (8)	0.0230 (6)	0.0105 (6)	0.0018 (5)	−0.0046 (6)
C14	0.0257 (6)	0.0414 (8)	0.0354 (7)	0.0009 (6)	0.0049 (5)	−0.0143 (6)
C15	0.0333 (7)	0.0308 (7)	0.0396 (7)	−0.0031 (6)	0.0110 (6)	−0.0035 (6)
C16	0.0307 (7)	0.0270 (7)	0.0268 (6)	0.0027 (5)	0.0063 (5)	0.0007 (5)
O1	0.0515 (6)	0.0275 (5)	0.0405 (5)	0.0123 (4)	0.0166 (4)	0.0030 (4)
O2	0.0320 (5)	0.0241 (5)	0.0214 (4)	0.0077 (4)	0.0021 (3)	0.0016 (4)

### Geometric parameters (Å, °)

**Table:**

C1—C2	1.5009 (16)	C8—C9	1.3828 (18)
C1—H1A	0.9800	C8—H8	0.9500
C1—H1B	0.9800	C9—C10	1.3839 (17)
C1—H1C	0.9800	C9—H9	0.9500
C2—O1	1.2165 (15)	C10—H10	0.9500
C2—C3	1.5089 (16)	C11—C16	1.3866 (16)
C3—C4	1.5403 (15)	C11—C12	1.3965 (15)
C3—H3A	0.9900	C12—C13	1.3855 (17)
C3—H3B	0.9900	C12—H12	0.9500
C4—O2	1.4266 (13)	C13—C14	1.3826 (19)
C4—C5	1.5301 (16)	C13—H13	0.9500
C4—C11	1.5373 (16)	C14—C15	1.3808 (18)
C5—C6	1.3830 (16)	C14—H14	0.9500
C5—C10	1.3964 (15)	C15—C16	1.3884 (17)
C6—C7	1.3934 (17)	C15—H15	0.9500
C6—H6	0.9500	C16—H16	0.9500
C7—C8	1.3795 (17)	O2—H2O	0.910 (12)
C7—H7	0.9500		
			
C2—C1—H1A	109.5	C6—C7—H7	120.0
C2—C1—H1B	109.5	C7—C8—C9	119.65 (12)
H1A—C1—H1B	109.5	C7—C8—H8	120.2
C2—C1—H1C	109.5	C9—C8—H8	120.2
H1A—C1—H1C	109.5	C8—C9—C10	120.33 (11)
H1B—C1—H1C	109.5	C8—C9—H9	119.8
O1—C2—C1	120.90 (12)	C10—C9—H9	119.8
O1—C2—C3	122.65 (11)	C9—C10—C5	120.69 (12)
C1—C2—C3	116.44 (11)	C9—C10—H10	119.7
C2—C3—C4	114.99 (10)	C5—C10—H10	119.7
C2—C3—H3A	108.5	C16—C11—C12	118.42 (11)
C4—C3—H3A	108.5	C16—C11—C4	121.49 (10)
C2—C3—H3B	108.5	C12—C11—C4	120.09 (11)
C4—C3—H3B	108.5	C13—C12—C11	120.70 (12)
H3A—C3—H3B	107.5	C13—C12—H12	119.6
O2—C4—C5	105.04 (8)	C11—C12—H12	119.6
O2—C4—C11	111.18 (9)	C14—C13—C12	120.15 (11)
C5—C4—C11	108.60 (9)	C14—C13—H13	119.9
O2—C4—C3	109.89 (9)	C12—C13—H13	119.9
C5—C4—C3	112.06 (10)	C15—C14—C13	119.72 (12)
C11—C4—C3	110.00 (9)	C15—C14—H14	120.1
C6—C5—C10	118.35 (11)	C13—C14—H14	120.1
C6—C5—C4	123.61 (10)	C14—C15—C16	120.17 (12)
C10—C5—C4	118.03 (10)	C14—C15—H15	119.9
C5—C6—C7	121.00 (11)	C16—C15—H15	119.9
C5—C6—H6	119.5	C11—C16—C15	120.84 (11)
C7—C6—H6	119.5	C11—C16—H16	119.6
C8—C7—C6	119.97 (12)	C15—C16—H16	119.6
C8—C7—H7	120.0	C4—O2—H2O	107.4 (8)
			
O1—C2—C3—C4	−15.52 (17)	C6—C5—C10—C9	0.08 (18)
C1—C2—C3—C4	165.07 (10)	C4—C5—C10—C9	−179.04 (11)
C2—C3—C4—O2	57.09 (13)	O2—C4—C11—C16	−2.22 (14)
C2—C3—C4—C5	173.47 (9)	C5—C4—C11—C16	−117.30 (11)
C2—C3—C4—C11	−65.63 (12)	C3—C4—C11—C16	119.73 (11)
O2—C4—C5—C6	133.63 (11)	O2—C4—C11—C12	177.02 (10)
C11—C4—C5—C6	−107.35 (12)	C5—C4—C11—C12	61.94 (13)
C3—C4—C5—C6	14.36 (15)	C3—C4—C11—C12	−61.03 (13)
O2—C4—C5—C10	−47.29 (13)	C16—C11—C12—C13	−0.53 (17)
C11—C4—C5—C10	71.72 (12)	C4—C11—C12—C13	−179.79 (11)
C3—C4—C5—C10	−166.56 (10)	C11—C12—C13—C14	−0.24 (18)
C10—C5—C6—C7	−0.05 (18)	C12—C13—C14—C15	0.74 (18)
C4—C5—C6—C7	179.02 (11)	C13—C14—C15—C16	−0.48 (19)
C5—C6—C7—C8	0.10 (19)	C12—C11—C16—C15	0.79 (17)
C6—C7—C8—C9	−0.17 (19)	C4—C11—C16—C15	−179.96 (11)
C7—C8—C9—C10	0.21 (19)	C14—C15—C16—C11	−0.29 (18)
C8—C9—C10—C5	−0.16 (19)		

### Hydrogen-bond geometry (Å, °)

**Table:**

*D*—H···*A*	*D*—H	H···*A*	*D*···*A*	*D*—H···*A*
O2—H2O···O1	0.910 (12)	2.016 (12)	2.7636 (12)	138.5 (11)
O2—H2O···O1^i^	0.910 (12)	2.385 (13)	3.0530 (12)	130.3 (10)

Symmetry codes: (i) −*x*+1, −*y*, −*z*+2.
